# Emergency Department Nurses’ Experiences in Caring for Older Adults With Dementia

**DOI:** 10.1093/geroni/igaa057.873

**Published:** 2020-12-16

**Authors:** YuJin Park, Eun-Hi Kong, Hyejin Kim

**Affiliations:** 1 Gachon Gil Hospital, Incheon, Republic of Korea; 2 Gachon University, Incheon, Republic of Korea; 3 Chung-Ang University, Seoul, Republic of Korea

## Abstract

The aim of this study was to describe emergency-department nurses’ experiences in caring for older adults with dementia. A qualitative descriptive method was employed. A convenience sampling method was used to recruit nurses from seven regional emergency medical centers. Data were collected through to in-depth interviews and field notes until data were saturated. A total of 23 emergency-department nurses participated in the first interview and 18 of them participated in the second interview. Data were analyzed using qualitative content analysis with the ATLAS. ti 8.2 software program. Through data analysis of interview data, 36 codes and 10 categories were identified. Finally, four themes emerged from data analysis: lack of preparation for dementia care, tired of caring for older adults with dementia, an inappropriate emergency-department environment for older adults with dementia, and needs for improvements in dementia care. Emergency-department nurses felt completely unprepared for dementia care and felt exhausted after caring for older adults with dementia. In addition, the emergency department is considered to be an environment that does not consider older adults with dementia and thus is unsuitable for older adults with dementia. Also, nurses require systematic support when caring for older adults with dementia and improvement in awareness in the emergency department. Study results provide important data to develop educational material for emergency care including criteria for emergency medical assessments and policies of emergency medical institutions. In addition, the results provide evidence for clinical practice guideline for nurses who care for older adults with dementia in the emergency departments.

